# Weeded Out? Gendered Responses to Failing Calculus

**DOI:** 10.3390/socsci6020047

**Published:** 2017-05-10

**Authors:** Tanya Sanabria, Andrew Penner

**Affiliations:** Department of Sociology, University of California, Irvine, Irvine, CA 92697, USA

**Keywords:** higher education, gender, STEM, inverse probability weighting

## Abstract

Although women graduate from college at higher rates than men, they remain underrepresented in science, technology, engineering, and mathematics (STEM) fields. This study examines whether women react to failing a STEM weed-out course by switching to a non-STEM major and graduating with a bachelor’s degree in a non-STEM field. While competitive courses designed to weed out potential STEM majors are often invoked in discussions around why students exit the STEM pipeline, relatively little is known about how women and men react to failing these courses. We use detailed individual-level data from the National Educational Longitudinal Study (NELS) Postsecondary Transcript Study (PETS): 1988–2000 to show that women who failed an introductory calculus course are substantially less likely to earn a bachelor’s degree in STEM. In doing so, we provide evidence that weed-out course failure might help us to better understand why women are less likely to earn degrees.

## 1. Introduction

A longstanding body of research on gender differences in education suggests that women are underrepresented in many science, technology, engineering, and mathematics (STEM) fields—particularly in the physical sciences and engineering (Xie and Shauman 2007). Research seeking to understand gender differences in who majors in a STEM field has identified a plethora of factors, ranging from discrimination, cultural stereotypes around gender and science, confidence, peer networks, and a preference for flexible curricula not offered in STEM departments (Correll 2001; Charles and Bradley 2009; Cech et al. 2011; Riegle-Crumb 2006; Mann and Diprete 2013). Underlying much of this research is the notion that STEM undergraduate training occurs in an environment that ranges from disengaging to competitive to chilly, and that this climate leads students to opt for other fields (Seymour and Hewitt 1997; Niederle and Versterlund 2007). While the factors that contribute to this climate are likewise numerous, competitive weed-out courses at the introductory level are a source of considerable dissatisfaction among undergraduates (Seymour and Hewitt 1997). These courses serve a gatekeeping function, as they are required for many STEM majors, and are often failed by a substantial number of students, promoting a competitive “sink or swim” environment (Seymour and Hewitt 1997; Kokkelenberg and Sinha 2010; Olson and Riordan 2012).

Importantly, both women and men see this as problematic. The women interviewed by Seymour and Hewitt express their thoughts like “I knew I could have done it if I wanted to. But I just said ‘Do you really want to do this? Is it really worth killing yourself for?’” or “It’s been unadulterated hell. Major overloads, no rest, stress—and it’s getting worse. That’s why I’m looking elsewhere” (Seymour and Hewitt 1997, pp. 202–3). Men’s assessments are largely similar: “I mean, why stay [in science]? You know, there’s no reason. And the rewards are—there’s no rewards. I mean, I can see no logical reason why you’d stay.” and “You go through hell in the sciences without any guarantee that you will be able to work. Why do it? Why not be an English major?” This sentiment is summarized by Meg Whitman, who noted in an interview that “I took calculus, chemistry, and physics my first year. I survived. But I didn’t enjoy it … After that, I had to find something else to do. I began selling advertising for a magazine that was published by Princeton undergrads. It was more fun than physics” (Fishman 2001).

However, despite the fact these weed-out courses are often invoked by students as a significant source of disengagement, surprisingly little is known about how undergraduates respond to failing these courses. While not examining weed-out course failure per se, research on grade inflation suggests that failing a weed-out class could play an important role in shaping students’ future majors. One study, for example, found that students were “pulled away” by their higher grades in the humanities, arts, and social sciences courses and “pushed out” of STEM because of lower grades (Ost 2010). Grade inflation in introductory classes may be particularly important, as the grades that students receive in introductory courses strongly predict whether students choose to enroll in more courses in the discipline (Ost 2010). Introductory courses in STEM departments tend to be among the lowest graded courses (Rask 2010). Simulations suggest that if the grading distribution in introductory science courses resembled the college average, there would be 2–4 percent increase in advanced science course taking in later semesters (Rask 2010).

We build on this research by examining whether there are gender differences in the rates at which men and women fail introductory calculus (which we henceforth refer to simply as calculus), and how they respond to failure. Calculus often serves a gatekeeping function across STEM disciplines, limiting the rate at which students can take advanced coursework in their major. Introductory math courses, such as calculus, were found to be important factors for students’ decisions to stay or switch out of STEM (Chen 2013). Although several studies have indicated that performance in introductory courses has been linked to STEM persistence, little attention has been given to failing weed-out courses like calculus. A key limitation in previous research is that these studies pool grades across STEM courses, using GPA as an indicator of poor performance. While important, these studies cannot ultimately address the role of weed-out course failure. Given the important signal that failing a weed-out course provides to students (Crisp et al. 2009), we argue that examining the gendered responses to calculus failure can provide researchers a better understanding of the critical junctures that shape a student’s academic trajectory.

Gender might play an important role in shaping how students respond to failing calculus given societal stereotypes about math competence. Correll (Correll 2004) shows that beliefs about gender differences in a domain can shape self-assessments of competence and interest in pursuing a career using these skills. Specifically, when women are exposed to the belief that men are superior in a particular domain, women rate their performance worse than men, even when men and women receive identical feedback about their actual performance in the domain. Given widespread stereotypes about gender differences in mathematics, Correll’s findings suggest that women who fail a calculus course might perceive their math skills to be worse than men who fail, and might have less interest in pursuing math-dependent careers. Gender differences in self-assessments driven by these stereotypes may explain why women tend to express doubts in their mathematical skills (Charles and Bradley 2009; Noel-Levitz 2014) and are more likely to switch to a female-typed major when receiving lower grades in coursework (Rask and Tiefenthaler 2008). As Charles and Bradley (Charles and Bradley 2009, p. 926) note, “Beliefs about gender difference can thus spawn powerful self-fulfilling prophesies”.

While previous research suggests that women are more likely to re-evaluate and change their career pathways in response to negative feedback, we know of no study that has examined the implications of calculus failure and gender differences on whether students major in STEM. This study uses a doubly robust inverse probability weighting approach to compare the degree outcomes of students who had taken and failed calculus to a comparison group who passed calculus. We thus provide the first examination of the potentially gendered ways in which students responded to failing weed-out coursework.

### Research Questions

Our key research question examines whether there are gender differences in the response to failing calculus, focusing on students’ likelihood of completing a bachelor’s degree, and in particular, on degree completion in a STEM field. To motivate the analyses for our central research question, we first ask (1) who takes and who fails calculus? Then, we ask, (2) what are the schooling outcomes associated with failing calculus? Finally, we address our key question, (3) are there gender differences in the schooling outcomes associated with failing calculus? To understand how failing a weed-out class may affect students in the STEM pipeline (i.e., those who may be considered at risk of majoring a STEM field), we narrow our sample size for questions (2) and (3) to students who planned to major in STEM as high school seniors.

## 2. Data

Data are from the National Education Longitudinal Study (NELS:88) and the NELS Postsecondary Education Transcript Study (PETS:2000) (NCES 1988; NCES 2000). The NELS:88 is a longitudinal study that followed a representative sample of 25,000 eighth-grade students over twelve years starting in 1988. The Educational Testing Service created pencil-and-paper tests to assess each eighth-grader’s skills in reading and mathematics for the NELS:88. These tests were repeated in tenth, and twelfth grades. We use the student’s percentile rank in the pencil-and-paper test in twelfth grade to measure students’ pre-college academic skills in reading and math.

During each follow-up survey, additional data and interviews were collected from parents, teachers, and students participating in the study. As a longitudinal panel study, NELS:88 experienced sample attrition and non-response bias. To adjust for the sampling frame, the NELS:88 replenished the sample with additional respondents. All analyses thus use weights to adjust for these differences and students in the analyses were non-missing in key outcome, predictor, and control variables.

The fourth and last follow-up study of NELS:88/2000 for the sample of the eighth-grade class of 1988 occurred in 2000. The study collected postsecondary education transcripts for the sample members who responded to the final follow-up and reported attendance at a postsecondary educational institution in the third (1994) or fourth (2000) follow-up. Approximately 16,020 postsecondary transcripts were collected for 15,240 sample members, a subsample from the third follow-up. Transcripts contained detailed information on students’ coursework, credits, grades, and degree obtained. To examine postsecondary education outcomes, we restricted our sample to the base-year through fourth follow-up studies, limiting the number of valid cases with a postsecondary transcript record to 7050 individuals.

## 3. Measures

Our key independent variable is failing an introductory calculus course, a key gate-keeping course that often serves as a requirement for STEM majors. Calculus courses were identified using the 2010 College Course Map (CCM) taxonomy system to code information on the course subject and title from college transcripts. Students were coded as having failed a class if they both (1) received a grade of “0” or “F” for the course and (2) reported zero earned credits for the course. We ran additional analyses where we define failure to include grades of “D”, “D-“, and “F”. Findings were consistent with results from analyses reported here.

The two main outcome variables in this study are whether a student completed a bachelor’s degree and whether they graduated with a bachelor’s degree in a STEM field. STEM majors include engineering, mathematics, physics, chemistry, and biology; a complete list of majors included as STEM fields is available in Appendix A (Table A1). The degree type and major is reported on the student’s transcript at collection.

We also control for a wide range of variables. Student-level controls include race/ethnicity, gender, socio-economic status, high school GPA (standardized), twelfth grade test score percentile ranks in both reading and math, whether students planned to major in STEM as high school students, and the highest math course taken while in high school. During students’ senior year of high school, students were asked if they expected to attend college and in which field they expected to major; we collapsed anticipated majors into an indicator for whether students planned to major in a STEM field. While we would ideally use a measure of intended major from the fall when students entered university, we prefer our measure from the senior year of high school to information collected in the third follow-up of NELS:88 in 1994, when most students were in their second year of college.

We also control for whether the student’s primary institution was a public two-year, private not-for-profit four-year, and a public four-year institution. Because some students move from one college to another, we coded for the first college that a student entered after high school. Accounting for observable differences on these dimensions helps ensure that the associations we observe between failing calculus and degree receipt are not being driven by these factors.

## 4. Sample

The first column of Table 1 provides a summary of the controls and outcome measures, as well as the number of students who took calculus and the number of students who failed (*n* = 3650). The study sample has slightly more women (52.6 percent) than men (47.4 percent). The sample consisted of primarily Non-Hispanic White (74.5 percent), with 7.5 percent identifying as Non-Hispanic Black, 11.5 percent identifying as Hispanic, and 6.6 percent as Asian. The average age that students entered college was 18.4, with ages ranging from 17 to 24.

To measure socioeconomic status, we use the socioeconomic status composite measure created by NELS, which combines information from the father’s education level, mother’s education level, father’s occupation, mother’s occupation and family income from the parent questionnaire data in NELS:88. In our sample, the average socioeconomic status (SES) composite is 0.08, meaning that the college-going students in our sample are relatively advantaged compared to the unweighted national average of −0.08 in NELS:88. For pre-college academic skills, we use the score percentile rank from the NELS pencil and paper test in reading and math that students took in twelfth grade in high school. On average, students in our sample of college-going students scored in the 60th percentile, meaning that students in our sample scored on average at the 60th percentile of the national distribution of high school seniors. The average high school grade point average (GPA) for our sample is 2.89. In our full study sample, about a quarter of students (24.9 percent) planned to major in STEM. We also take into account the highest level of mathematics course taken in high school, creating a series of indicators for whether students’ highest math course was Algebra I or similar (10 percent), geometry (13 percent), Algebra II (34 percent), Trigonometry (15 percent), pre-Calculus (16 percent), or Calculus (12 percent).

Looking at institution-level characteristics, we see that approximately 38 percent of the students in our sample entered a public two-year institution as their primary institution, while 18 percent entered a private not-for-profit four-year institution, and around 45 percent entered a public four-year institution. Approximately 15 percent of the entire sample had taken calculus and 1.6 percent of the entire sample (10.7 percent of calculus takers) had failed calculus. Regarding key outcomes, about less than half of the sample (41 percent) had earned a bachelor’s degree in any field as of 2000, while 46 percent did not. About 13 percent of the sample received a bachelor’s degree in a STEM field.

The second and third sets of columns of Table 1 provide the summary of covariates, outcome measures and independent variables among students who planned to major in STEM (*n* = 910) and those who did not plan to major in STEM (*n* = 2740), respectively. The group of students who planned to major in STEM is more evenly split by gender (49 percent men and 51 percent women) compared with the group of students who did not plan to major in STEM (47 percent men and 53 percent women). There are fewer White students (70 percent as compared with 76 percent), more Black students (10 percent as compared with 7 percent), fewer Hispanic students (11 percent compared with 12 percent), and more Asian students (9 percent as compared with 6 percent) in the group of students who planned to major in STEM. Students who planned to major in STEM demonstrate slightly higher levels of pre-college academic skills (scoring on average at the 63rd percentile compared with the 60th percentile) and achievement (2.98 GPA compared with 2.86) than those who did not plan to major in STEM fields. A significantly larger proportion of students who planned to major in STEM had taken Calculus as their highest math course in high school (20 percent) compared to those who did not plan to major in STEM (10 percent) while a higher proportion of students who did not plan to major in STEM fields had taken up to Algebra II (36 percent compared with 29 percent).

The percentages of students who entered a public two-year, a private not-for-profit four-year, or a public four-year institution as their primary institution in each group were fairly similar to the full sample. Approximately 28 percent of students who planned to major in STEM took calculus in college compared to 11 percent of students who did not plan to major in STEM. Four percent of students who planned to major in STEM as high school seniors had failed calculus, while one percent of students who did not plan to major in a STEM field failed calculus. The percentages of students who earned a bachelor’s degree in each group were fairly similar to the full sample. Approximately 28 percent of students who planned to major in STEM earned a bachelor’s degree in a STEM field, while 8 percent of students who did not plan to major in STEM earned a STEM bachelor’s degree.

## 5. Methods

### Estimation Strategy

We use doubly robust inverse probability weighting (IPW) to examine the relationship between failing calculus and degree outcomes among calculus takers. In our observational data, we cannot randomly assign our treatment (e.g., calculus failure). As such, students who fail calculus are likely to be different from those who did not fail calculus (our “control” condition) in both observable and unobservable ways. Table 2 provides descriptive results on students who take and fail calculus in the study sample. We see in Table 2 that there are both demographic and institutional differences between students who pass and students who fail calculus. Given these differences, we cannot estimate the effect of calculus failure on degree completion by simply comparing the estimates of degree completion likelihood among those who failed or students who passed calculus. To address this issue, we use IPW estimates to account for differences in the observable characteristics of students who pass and fail calculus.

IPW estimators use a two-step approach. First, the predicted probability of receiving the treatment is estimated for each student. Then, weights for each student are created. To balance the groups on observable characteristics, the IPW scheme up-weights students who received a given treatment but were unlikely to receive the treatment based on observable characteristics (e.g., students who were likely to fail but passed, or who were likely to pass but failed). Conversely, the scheme down-weights students who were highly likely to receive the treatment they received.

One limitation of IPW is that it assumes that the model used to predict the treatment (and therefore the weight) is correctly specified. If this model is not correctly specified, then the weighting will not account for the differences in these observable characteristics. We can relax the model specification assumption by using doubly robust IPW estimators and include controls in our weighted models predicting our outcomes. In these models, if either the weighting model or the final model is correctly specified, we will account for potential imbalance in our observable characteristics. It is important to clarify, however, that doubly robust models do not account for differences in unobserved characteristics of respondents. For a step-by-step process of how we created the doubly robust IPW estimators, see Appendix B.

## 6. Results

### 6.1. Predicting Calculus Taking and Performance

Table 2 presents the results of linear probability models in order to provide descriptive information on the characteristics of students who (a) take calculus compared to the entire study sample (*n* = 3490) and (b) fail calculus compared to students who had passed calculus (*n* = 540).

Model 1 shows that women are 11 percentage points less likely to take calculus than men, and that Asian students are nine percentage points more likely to take calculus than white students. A one-unit increase in SES composite is associated with a two percentage-point increase in taking calculus. One percent increases in students’ reading and math scores and high school GPA are associated with four and 11 percentage-point increases in the likelihood of taking calculus, respectively. Compared to students who had algebra I or a similar course as their highest math class in high school, students who took algebra II are, if anything, slightly less likely to take calculus, while students who took calculus in high school were 31 percentage points more likely to take calculus in college. Students who planned to major in STEM as high school seniors were 13 percentage points more likely to take calculus. Finally, students entering a four-year private or public college (compared to entering a two-year college) were six and three percentage points more likely to take calculus, respectively.

Model 2 examines how the same set of factors from Model 1 are associated with failing calculus among students who took it. Importantly for our purposes, we see no gender differences in the likelihood of failing among calculus takers. We do find that high SES students, as well as students with higher GPAs in high school are less likely to fail. We also find that students who directly enter a four-year college are more likely to fail than students who first entered a two-year college. All other variables in the model yielded statistically non-significant findings.

### 6.2. General and STEM Bachelor Degree Attainment

Our results examining the relationship between failing calculus and degree attainment are presented in Table 3. As noted earlier, to focus on students who might plausibly be in the STEM pipeline, we restrict our analyses here to students who (a) planned to major in STEM in their senior year of high school and (b) had taken calculus in college. Students in this sample were weighted based on their probability of being assigned to treatment received. To address concerns around misspecification in the weighting model, we estimate doubly robust models that include all covariates in the models predicting our outcomes. In the first two models, we first examine whether students completed a bachelor’s degree in any field. Models 3 and 4 examine whether students attained a bachelor’s degree specifically in a STEM field.

**Figure 1:**
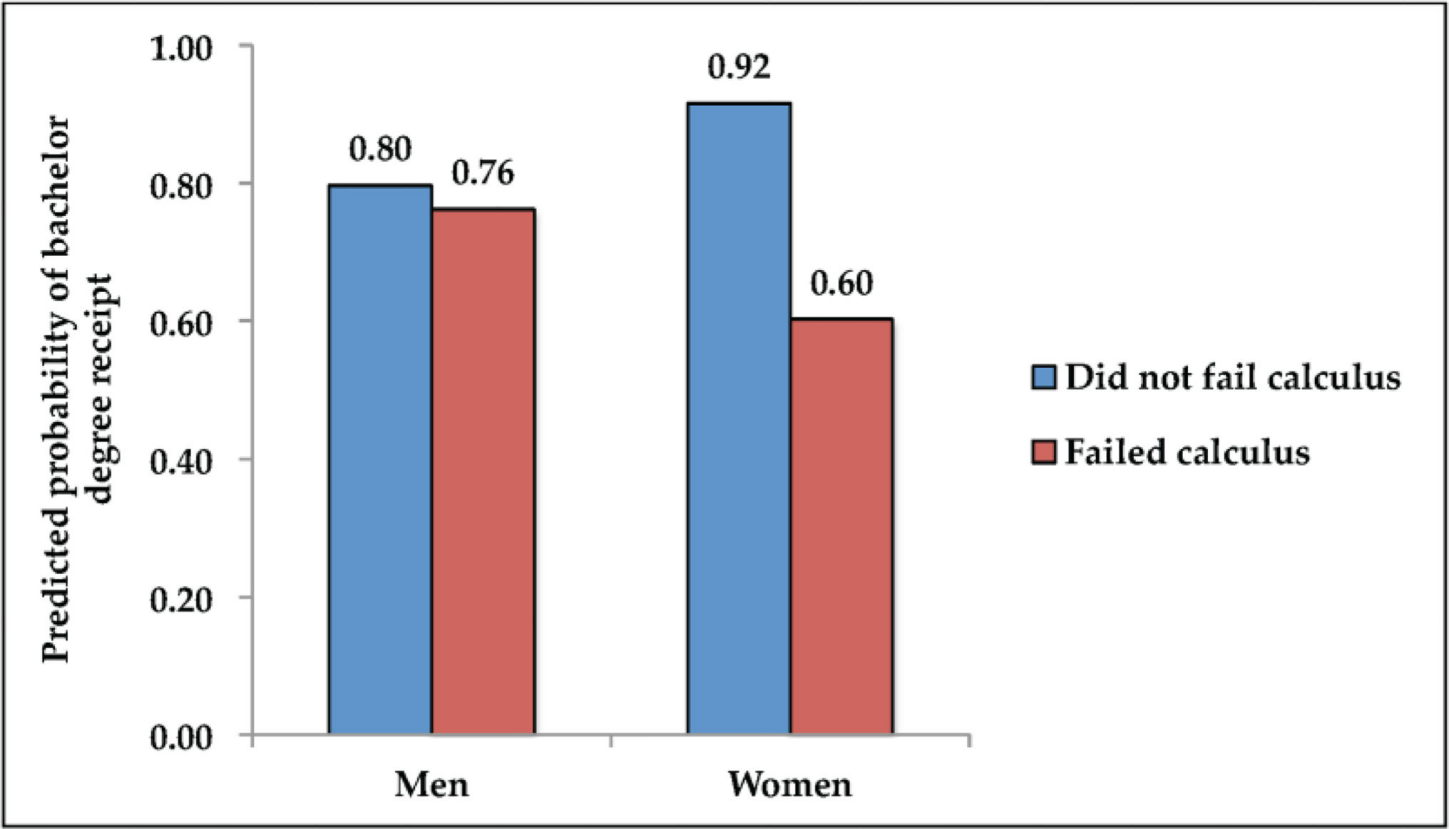
Predicted probabilities of bachelor degree receipt by gender. Source: National Educational Longitudinal Study (NELS:88) and Postsecondary Education Transcript Study (PETS:2000) (NCES 1988; NCES 2000).

In Model 1, we examine the relationship between failing calculus and completing a bachelor’s degree. After accounting for demographic characteristics, prior achievement, academic skill, highest math course taken in high school, and institution-level covariates, we find that failing calculus is associated with a 12 percentage-point decrease in degree completion. In Model 2, we interact failing calculus and gender to see whether the relationship between failing calculus and bachelor degree completion varies by gender. To facilitate interpretation, we present predicted probabilities from Model 2 (holding covariates constant so that covariates are averages for the study sample) in Figure 1. While we find only small differences in the likelihood of receiving a bachelor’s degree between men who passed and failed calculus (0.80 versus 0.76), we see that women who did not fail calculus are 32 percentage points more likely to receive a bachelor’s degree than women who failed calculus (0.92 versus 0.60; *p* = 0.019). Men’s likelihood of receiving a bachelor’s degree is thus not strongly tied to whether they pass calculus, while for women it is. Women who pass calculus are more likely to get a bachelor’s degree than men, while women who fail calculus are less likely to do so.

**Figure 2:**
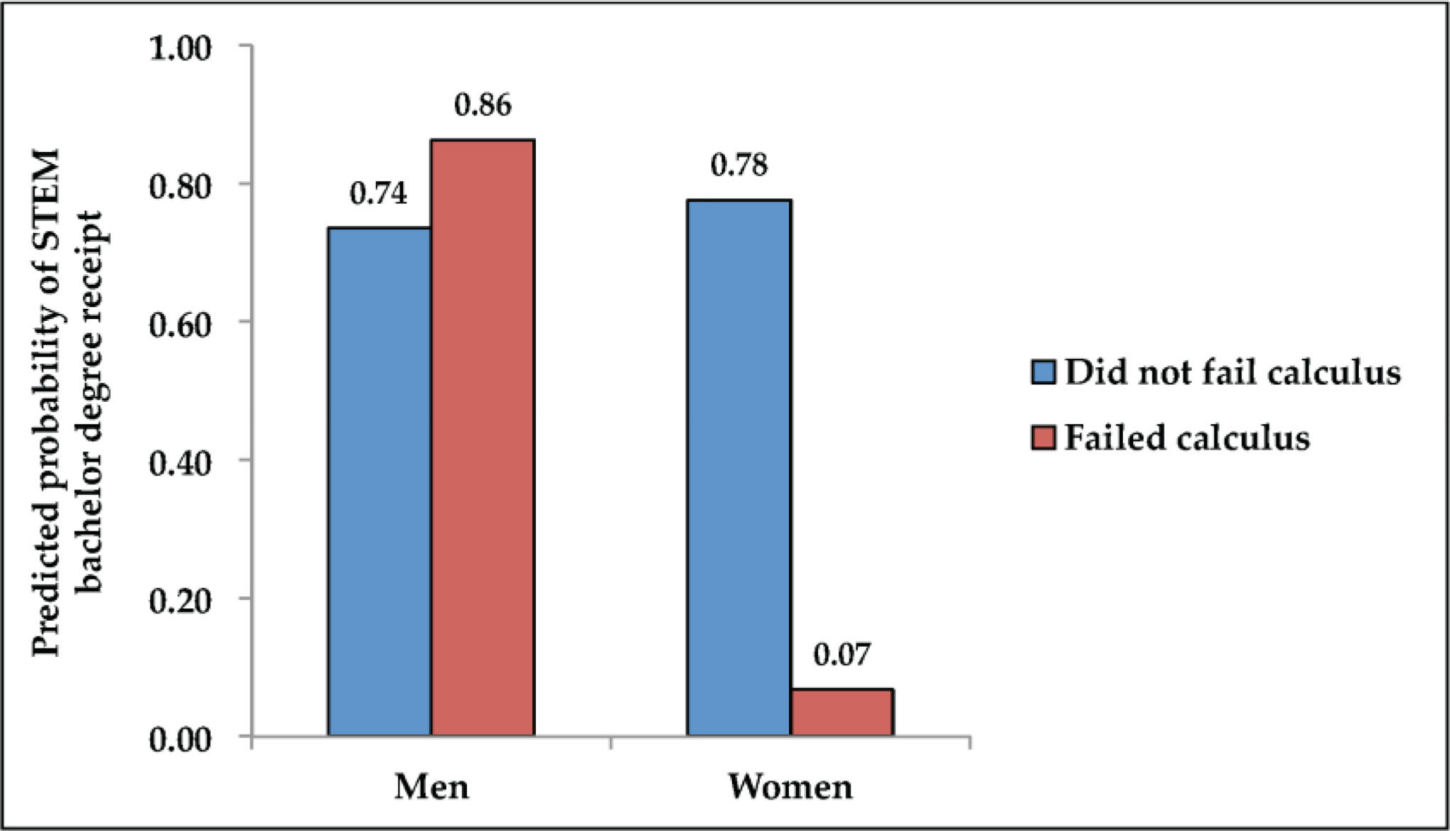
Predicted probabilities of bachelor degree receipt in a Science, Technology, Engineering, and Mathematics (STEM) field by gender. Source: National Educational Longitudinal Study (NELS:88) and Postsecondary Education Transcript Study (PETS:2000) (NCES 1988; NCES 2000).

Model 3 in Table 3 examines the relationship between failing calculus and STEM bachelor’s degree completion. Here we find that, overall, failing calculus was not statistically significant (*p* = 0.165), though the point estimate is similar in magnitude and direction as in Model 1, suggesting that students who fail are less likely to obtain a STEM degree. Model 4 follows Model 2, examining the relationship between failing calculus and receiving a STEM bachelor’s degree by gender. Predicted probabilities from Model 4 are reported in Figure 2. As above, we find no statistically significant differences among men (0.74 versus 0.86), but we do find that there is a statistically significant difference between women who do and do not fail (0.07 versus 0.78, *p* < 0.001). As is readily visible in Figure 2, failing calculus does not appear to weed out men, but does appear to weed women out.

## 7. Discussion

Despite widespread interest in the role of weed-out classes in the STEM training pipeline, little is known about how failing a weed-out class might shape both men and women’s STEM decisions to major in a STEM field. Using nationally representative data and a wide range of controls, we find that women who intended to major in STEM and fail calculus in college are significantly less likely to obtain a bachelor’s degree in a STEM field. For men who intend to major in a STEM field, on the other hand, we find no evidence that failing calculus lowers their likelihood of obtaining a STEM degree. To the degree that calculus functions as a weed-out class, our findings suggest that it does so in a profoundly gendered way, weeding out women but not men.

Our results have important consequences for policies aimed at increasing the representation of women in STEM fields. Given that calculus often serves as a gatekeeper for advanced courses in STEM, students who fail calculus face additional barriers that make it difficult to continue with their college studies in many STEM fields (Seymour and Hewitt 1997; Chen 2013). Our findings suggest that these barriers do little to dampen men’s STEM degree completion, but may play a substantial role in shaping women’s STEM degree completion. Policies aimed at increasing the representation of women obtaining STEM degrees may want to focus on women at this crucial stage, and efforts to assist students who have failed calculus may want to focus particularly on women. More broadly, given the lack of an effect on men’s majors, these findings suggest that STEM educators may want to rethink the role of weed-out classes in STEM education. That is, it is difficult to argue that weed-out classes are doing their job and keeping unprepared individuals from pursuing these majors, when men who fail calculus are just as likely to graduate with a STEM degree as men who pass.

This lack of a difference for men is perhaps puzzling and raises additional questions. For example, it is unclear at what rate we would want men and women who failed calculus to continue pursuing STEM degrees (Penner and Willer 2015). Women are generally more responsive to grades than men (Charles and Bradley 2009), and while research on STEM persistence typically operates under the assumption that STEM persistence should be encouraged for all individuals, it seems plausible that after failing a weed-out class, pursuing a different major is potentially more adaptive than continuing to major in STEM. That is, while qualities like grit (Duckworth et al. 2007) and resilience (Masten 1994) are rightfully celebrated, adaptive goal disengagement (Heckhausen and Schulz 1995) is also an important adaptive strategy. To use a non-educational example, somebody who has repeatedly asked a romantic interest to go on a date and been turned down should potentially disengage from the goal of being in a romantic relationship with this individual, rather than continue to persist. While we are unable to adjudicate whether the women who fail weed-out classes are best served by persisting in STEM fields, we argue that understanding the outcomes associated with weed-out class failure provides insight into the larger structural changes needed to alter students’ persistence decisions.

In line with arguments around adaptive goal disengagement, our findings could in part also reflect the fact the women who fail calculus have better non-STEM options than men (Penner 2015; Wang et al. 2013). If this was the case, weed-out classes could plausibly explain both why women are less likely to major in STEM fields (they switch their majors after failing) and why men are less likely to graduate from college, net of enrollment rates (if they drop out after failing a weed-out class). As we only find evidence for the first of these processes, this suggests a gendered dimension in how calculus weeds women out of STEM fields. It also seems unlikely that these differences could produce differences of the magnitude we observe here. However, this perspective does highlight that we should not view women dropping out of the STEM pipeline as failures, but instead focus on questions around how STEM fields are structured.

In addition to questions about the larger structure of STEM education, larger societal stereotypes about gender and STEM are potentially relevant. One explanation for our findings is that the weed out culture for introductory-level coursework combines with gendered stereotypes about STEM fields to result in different self-assessments after calculus failure (Correll 2004). That is, much like the women in Correll’s study who expressed less interest in pursuing fields that were said to be male advantaged, larger gender stereotypes might shape how women who fail calculus incorporate this information into their self-assessments and interests differently than men.

In supplemental analyses, we considered whether failure in any course deters women from earning a STEM degree. Taking a sample of students in the humanities “pipeline,” we estimated whether failing introductory writing composition is more likely to deter women than men from graduating with a humanities degree using the same IPW estimation strategy described above. While failing introductory writing is negatively associated with completing a bachelor’s degree and a humanities bachelor’s degree, we find no gender differences in humanities degree attainment rates among those who failed this course. We also examined other potential STEM weed-out courses (e.g., introductory chemistry), and do not find similar patterns in these courses as for calculus. This is perhaps surprising, and may speak to the unique space that calculus occupies.

## 8. Limitations

While we provide important evidence regarding the different ways in which women and men respond to failing weed-out courses, our study has several limitations. The first is the possibility that students who have failed calculus are different from students who did not in unobservable ways, limiting causal attributions. While we account for a wide range of observable characteristics by estimating doubly robust IPW, our approach cannot account for unobserved differences between the students who did and did not fail calculus.

Another limitation of our study is our lack of information about students’ intended majors before and after taking calculus. We use information about whether students planned to major in STEM as high school seniors to indicate whether students could be in the STEM pipeline at this point, but cannot isolate failing calculus as being the factor that led students to pursue a different major. For example, we lack information on other important factors associated with college and STEM persistence, such as quality of faculty-student contact in the STEM department, peer interactions, experiences or perceptions of diversity on the college campus, student satisfaction, and participation in extracurricular activities while enrolled in college (Seymour and Hewitt 1997). Of particular note, we lack data on perceptions of failure, motivation, and self-efficacy in the NELS:88 (Tinto 1987). However, to the degree that many of these considerations could be mediators that helped explain why failing mattered, it is unclear that they should be introduced as control variables. Additionally, while we acknowledge that calculus takers across STEM majors may differ, the limited sample size in our study does not allow separating out analyses by specific major (e.g., physical versus biological sciences).

Finally, although we use a large, nationally representative dataset to examine these questions, the number of individuals who intended to major in a STEM field and took (and failed) calculus is relatively small, necessitating caution in interpreting the results. As such, these results would benefit from future replication studies. Furthermore, as noted above, in our supplemental analyses, we find evidence suggesting that calculus may be unique, as we do not find similar patterns for other introductory STEM courses. However, given the relatively small samples for these classes, future work on this question would be particularly useful in understanding if other attributes to its position in the course sequence, course content, pedagogy or other factors play a role in weeding out women but not men. In particular, while we focus on calculus, given its prominent position and relative prevalence, future work might fruitfully examine whether other weed-out classes function in similar gendered ways.

## 9. Conclusions

Gender disparities in postsecondary STEM education continue to be an enduring issue in higher education. Our study examined how men and women react differently to failing a weed-out course among potential STEM majors, which might shape their educational pathways. Using detailed individual-level data from NELS PETS:1988–2000, we find that women who planned to major in STEM and failed calculus in college were substantially less likely to obtain a bachelor’s degree in STEM. On the other hand, failing calculus did not appear to lower the likelihood of STEM degree receipt among men. Thus, we demonstrate evidence of the gendered ways these weed-out courses function—weeding out women but not men in the STEM degree pipeline.

## Figures and Tables

**Table 1:** Descriptive statistics of variables used in analyses (*n* = 3650).

	Full Study Sample		Planned to Major in STEM		Did Not Plan to Major in STEM	
	
	# valid obs	mean/%	# valid obs.	mean/%	# valid obs.	mean/%
	3650		910		2740	

**Gender**						

Male	1730	47.4%	450	49.5%	1280	46.7%
Female	1920	52.6%	460	50.5%	1460	53.32%

**Race/Ethnicity**						

White (Non-Hispanic)	2720	74.5%	640	70.5%	2080	75.8%
Black (Non-Hispanic)	270	7.5%	90	10.2%	180	6.6%
Hispanic	420	11.5%	100	10.6%	320	11.8%
Asian	240	6.6%	80	8.7%	160	5.8%
**Age when entered college**	3650	18.4	910	18.3	2740	18.4
**Socioeconomic status (composite)**	3650	0.08	910	0.04	2740	0.09

**Prior Ability and Achievement**						

NELS test score percentile	3650	60.6	910	62.6	2740	60.0
High School GPA	3650	2.89	910	2.98	2740	2.86

**Highest Math Course Taken in High School**						

Algebra I or equivalent	380	10.3%	80	8.%	300	11.0%
Geometry	480	13.2%	100	11.0%	380	13.7%
Algebra II	1250	34.2%	260	28.5%	990	36.1%
Trigonometry	550	15.1%	130	14.3%	420	15.3%
Pre-calculus	570	15.6%	170	18.7%	400	14.6%
Calculus	430	11.8%	180	19.8%	260	9.5%

**Primary Institution Type**						

Public 2 year	1380	37.8%	340	37.3%	1040	38.0%
Private Not-For Profit 4-year	640	17.5%	150	16.5%	490	17.9%
Public 4-year	1630	44.7%	430	47.3%	1200	43.8%

**Planned to Major in STEM**						

Did not plan to major in STEM	2740	75.1%	–	–	–	–
Planned to major in STEM	910	24.9%	–	–	–	–

**Calculus Course**						

Taken calculus	560	15.3%	250	27.5%	300	11.0%
Failed calculus	60	1.6%	40	4.4%	30	1.1%

**Degree Attainment**						

Earned a bacherlor‘s degree	1510	41.4%	360	39.6%	1150	42.0%
Did not earn a bacherlor‘s degree	1660	45.5%	400	52.7%	1260	46.0%

**Earned a Bachelor‘s in STEM**						

Did not earn bacherlor‘s degree in STEM	1190	32.6%	150	16.5%	1050	38.2%
Earned bacherlor‘s degree in STEM	470	12.9%	250	27.5%	220	8.0%

Source: National Educational Longitudinal Study (NELS:88) and Postsecondary Education Transcript Study (PETS:2000) (NCES 1988; NCES 2000). Sample restricted to students who had valid non-missing information on their postsecondary enrollment status, coursework, institution type, gender, race, age, NELS 12th grade test score percentile, high school GPA, highest math course taken in high school, and orientation towards majoring in a science, technology, engineering or mathematics (STEM) field in college. Degree attainment does not include students who earned an Associate’s Degree. *n* in models have been rounded to the nearest 10 for disclosure.

**Table 2:** Linear Probability Models (LPM) predicting who takes calculus and who fails calculus.

	Taken Calculus	Failed Calculus
	
	Compared to Students Who Never Took Calculus	Only among Students Who Took Calculus
*Demographics*		
Female	−0.11 *** (−8.20)	−0.02 (−0.44)
Age	−0.38 (0.11)	−0.76 (−1.46)
Age squared	0.01 (0.11)	0.02 (1.50)
Black	0.01 (0.68)	−0.01 (−0.81)
Hispanic	0.01 (0.60)	0.01 (0.15)
Asian	0.09 * (2.31)	0.07 (0.84)
Socio-economic status composite	0.02 * (2.21)	−0.06 * (−2.11)
*Prior academic skills and achievement*		
NELS 12th grade test score percentile (logged)	0.04 *** (4.69)	−0.03 (−0.45)
High school GPA (logged)	0.11 *** (4.27)	−0.17 + (−1.70)
*Highest math course taken in High School*		
Geometry	−0.03 (−1.64)	−0.20 (−1.06)
Algebra II	−0.02 + (−1.76)	0.07 (−0.35)
Trigonometry	0.04 + (1.90)	−0.07 (−0.37)
Pre-calculus	0.10 *** (3.77)	−0.11 (−0.59)
Calculus	0.31 *** (8.75)	−0.12 (−0.65)
Planned to major in STEM	0.13 *** (7.23)	0.03 (0.74)
*Institution Type*		
Private not-for-profit 4-year	0.06 ** (2.62)	0.05 (1.23)
Public 4-year	0.03 * (2.18)	0.11 * (2.37)
Constant	3.37 (7.23)	7.53 (1.55)
*R^2^*	0.24	0.11
*n*	3490	540

Source: National Educational Longitudinal Study (NELS:88), Postsecondary Education Transcript Study (PETS:2000) (NCES 1988; NCES 2000). *t*-statistics underneath coefficients in parentheses. Controls are in reference to male, White, highest math course taken as Algebra I or other math course in high school, and entered a public two-year college. Sampling weight used in analyses. *n* in models have been rounded to the nearest 10 for disclosure.

+ *p* < 0.1,

* *p* < 0.05,

** *p* < 0.01,

*** *p* < 0.001.

**Table 3:** Linear Probability Models (LPM) predicting receipt of a bachelor’s degree and receipt of a bachelor’s degree in a STEM field, among students who had taken calculus and planned to major in STEM.

	Bachelor’s Degree	Bachelor’s Degree	STEM Bachelor’s	STEM Bachelor’s
Failed calculus	−0.12 + (−1.66)		−0.12 (−1.39)	
*Gender and Failure Status* (Omitted category: men—did not fail calculus)				
Men—failed calculus		−0.03 (−0.34)		0.13 (1.30)
Women—did not fail calculus		0.12 + (1.82)		0.04 (0.48)
Women—failed calculus		−0.19 (−1.45)		−0.66 *** (−7.40)
Constant	16.43 (0.67)	18.14 (0.76)	−52.37 (−1.52)	−44.35 (−1.36)
*R^2^*	0.25	0.27	0.31	0.42
*n*	230	230	190	190

Source: National Educational Longitudinal Study (NELS:88) and Postsecondary Education Transcript Study (PETS:2000) (NCES 1988; NCES 2000). STEM in reference to science, technology, engineering or mathematics fields. *t*-statistics underneath coefficients in parentheses. Reference category for interactions is a male college student who did not fail calculus. Includes demographic, prior achievement/academic skills, and institution controls for doubly robust estimates. *n* in models has been rounded to the nearest 10 for disclosure.

+ *p* < 0.1,

* *p* < 0.05,

** *p* < 0.01,

*** *p* < 0.001.

## Appendix A

**Table A1:** Coding for Expected Majors and Received Majors as STEM.

Planned to Major in STEM	Did Not Plan to Major in STEM
Architecture and Related Programs	Agricultural Business and Production
Biological and Life Sciences	Area, Ethnic and Cultural Studies
Computer and Information Sciences	Business Management
Engineering	Communications
Engineering Related Technologies	Education
Mathematics	Health Professions
Physical Sciences	Humanities
Science Technologies	Law
	Liberal Arts and Sciences
	Public Administration and Services
	Reserve Officers’ Training Corp (R.O.T.C)
	Social Sciences
	Vocational Education
	Visual and Performing Arts

## Appendix B

### Doubly Robust Inverse Probability Weighting

In the first step of doubly robust IPW, we estimate propensities (*P*) for each student. Using covariates discussed earlier, each student is given a propensity score. An individual variable does not have to be a statistically significant predictor of treatment in the propensity model since the objective is for students in the treated and control categories to be balanced on the covariates. The propensity score equation is a logit model predicting the probability of a student receiving an F in calculus. All individual-level and college-level covariates discussed above were included in the logistic regression equation to predict the probability of treatment:

(A1) $$\text{Pr}{\left(\text{Fail}\right)}_{i}=\alpha_{i}+\beta_{k}X_{\text{ki}}+\varepsilon_{i}.$$

Equation (A1) predicts the probability of a student failing calculus in college and **X**_i_ is a vector of control variables. In the model above, *i* represents the value of an individual in the predictor equation.

After estimating each student’s predicted probability of failing calculus in Equation (A1), we then use the probabilities to create inverse probability weights, which we define as the inverse of the probability of receiving or not receiving the treatment given observable characteristics. For students at each category of treatment *t* (failed or passed calculus), we define our inverse probability weight as:

(A2) $$W=1/{\hat{P}}_{t},$$

where *P̂_t_* is the predicted probability that a student received the treatment that he or she received.

For doubly robust IPW estimators, the same covariates used to estimate the probability weights for Equation (A1) are also included as controls in a linear probability model predicting our degree outcomes. To examine whether the relationship between failing calculus and degree outcomes vary by gender, we estimate models that interact failing calculus with gender. We estimate two sets of these models; the first set predicts bachelor degree completion in any field and the second set predicts STEM bachelor degree completion. Thus, our first model in Table 3 predicts whether students completing a bachelor’s degree in any field as a function of failing calculus:

(A3) $$\text{Pr}{\left(\text{Degree}\right)}_{i}=\alpha_{i}+\beta_{1}{\text{Fail}}_{i}+\beta_{k}X_{\text{ki}}+\varepsilon_{i},$$

where *Fail_i_* is a dummy variable equal to one if a student ever failed calculus and zero otherwise and **X**_i_ is a vector of background controls for doubly robust estimates. The main effect of *Fail_i_* provides information about the association between failing calculus and receiving a bachelor’s degree. In the next model, we include an interaction effect between *Fail*_i_ and whether the student was female to examine the association any variation between failing calculus and bachelor degree completion by gender. The error term, *ε_i_*, captures characteristics not accounted for in the model that influences the outcome variable. We estimate similar models predicting STEM bachelor degree receipt.
